# Efficacy of sulphadoxine-pyrimethamine + artesunate, sulphadoxine-pyrimethamine + amodiaquine, and sulphadoxine-pyrimethamine alone in uncomplicated falciparum malaria in Mali

**DOI:** 10.1186/s12936-015-0557-y

**Published:** 2015-02-07

**Authors:** Hamma Maiga, Abdoulaye A Djimde, Abdoul H Beavogui, Ousmane Toure, Mamadou Tekete, Cheick Papa O Sangare, Antoine Dara, Zoumana I Traore, Oumar B Traore, Souleymane Dama, Christelle N’Dong, Hamidou Niangaly, Nouhoum Diallo, Demba Dembele, Issaka Sagara, Ogobara K Doumbo

**Affiliations:** Molecular Epidemiology and Drug Resistance Unit, University of Sciences Techniques and Technology of Bamako, PO Box: 1805, Bamako, Mali

**Keywords:** *Plasmodium falciparum*, Sulphadoxine-pyrimethamine, Non-artemisinin-based combination therapy, ACT, Mali

## Abstract

**Background:**

*Plasmodium falciparum* resistance to artemisinin has been reported in South-East Asia. Long half-life drugs are increasingly being used for malaria prevention. The potential spread of parasite resistance to these regimens is real and makes regular efficacy surveillance a priority.

**Methods:**

From August to December 2004 and July to December 2005, a randomized open label trial of sulphadoxine-pyrimethamine (SP) + artesunate (AS) *versus* SP + amodiaquine (AQ), and SP alone, was conducted in two villages of Mali. PCR was used to distinguish new infections from recrudescent *P. falciparum* infections. Patients were followed for 28 days to assess treatment efficacy.

**Results:**

Overall 912 children aged between six to 59 months, with uncomplicated *P. falciparum* malaria were recruited. Baseline characteristics were similar in the three treatment arms. Crude ACPRs were 94.9%; 98.6% and 93.5% for SP + AS; SP + AQ and SP alone arms respectively (SP + AS *versus* SP + AQ, p = 0.01; SP + AS *versus* SP, p = 0.5; SP + AQ *versus* SP, p = 0.001). After PCR adjustment, cACPRs were 99%; 100% and 97.2% for SP + AS; SP + AQ and SP alone arms, respectively (SP + AS *versus* SP + AQ, p = 0.25; SP + AS *versus* SP, p = 0.12; SP + AQ *versus* SP, p = 0.007).

**Conclusion:**

Sulphadoxine-pyrimethamine + amodiaquine therapy was as efficacious as sulphadoxine-pyrimethamine + artesunate, but more efficacious than sulphadoxine-pyrimethamine alone in the treatment of uncomplicated *P. falciparum* malaria in Mali.

## Background

The emergence of *Plasmodium falciparum* resistance to chloroquine (CQ) has challenged malaria control efforts. Previous studies indicated that CQ resistance rates were above 25% in several sites of Mali [1-3]. These data compelled the Ministry of Health to change to artemisinin-based combination therapy (ACT) as first-line treatment of uncomplicated malaria, as recommended by the World Health Organization (WHO) [4]. The two ACT recommended by the Malian National Malaria Control Programme were artesunate-amodiaquine (AS + AQ) and artemether-lumefantrine (AL), which were both found to be efficacious and well-tolerated in the country [5,6]. However, because of uncertainties in the availability of these first line ACT in those early days of the ACT era there was a need for local data on the safety and efficacy of non artemisinin-based combinations. In addition, the emergence of artemisinin resistance would severely limit treatment options. Amodiaquine (AQ) and sulphadoxine-pyrimethamine (SP) were readily available in monotherapies and constituted attractive alternatives. This is even more pertinent with the recent reports from Asia were artemisinin resistance has been confirmed [7,8] and may already be spreading [9,10].

A nationwide effort of implementation of ACT was ongoing by the Malian National Malaria Control Programme with support from several partners. WHO recommendations allowed the use of non-artemisinin combination treatment regimens for the treatment of uncomplicated malaria in settings, where ACT was not available and the component drugs were efficacious and well tolerated [11]. In Mali, SP was still efficacious [2,12] and was restricted to intermittent preventive treatment of malaria during pregnancy (IPTp) [13]. Similarly, AQ was shown to be efficacious as a monotherapy in Mali [14]. At the initiation of this study, the combination of SP + AQ was contemplated as a potential combination to be used as an alternative to ACT as well as for intermittent preventive treatment of malaria in children (IPTc). An *in vivo* efficacy trial was designed to evaluate this non-artemisinin-based combination and compare it with SP + artesunate (AS), and SP alone.

## Methods

### Study site and population

The trial was conducted in two malaria hyper endemic rural villages, Kolle and Bancoumana, both located 57 to 60 kilometers southwest of Bamako, Mali. Data were collected in Kolle and Bancoumana Heath centers, respectively. Falciparum malaria is endemic and seasonal with parasitaemia prevalence ranging from 40–50% in the dry season (October-May) and 70–85% in the rainy season (June-September) [15]. The main vectors were *Anopheles gambiae sensu lato* (95.5% of vector population) and *Anopheles arabiensis* (4.5%). The mean monthly entomologic inoculation rate was 2.8 infectious bites per person, with marked seasonal variations [16].

### Study design and procedures

The Ethic Committee of the Faculty of Medicine, Pharmacy and Odonto-Stomatology, University of Bamako approved the study protocol. Permission was also obtained from community and local authorities. Informed consent was obtained from parents or infant guardians, according to previously described procedures [17]. Briefly, individual written informed consent was obtained from a parent or guardian of each child prior to inclusion. Two copies of consent documents were signed; one was kept with the study team and the second one was given to the parent or guardian. If a parent could not read, the consent was obtained in the presence of a witness who also signed the consent form.

The study was a randomized open label clinical trial conducted between August and December 2004 and July and December 2005. Patients were enrolled if they were aged between six and 59 months and fulfilled the following WHO *in vivo* criteria [18]: i) microscopy-diagnosed mono-infection of *P. falciparum* with a parasitaemia of 2,000 – 200,000/μl; ii) axillary temperature of ≥ 37.5°C; iii) haemoglobin ≥ 5 g/dL; iv) absence of febrile illness caused by diseases other than malaria, and v) absence of danger signs (inability to stand or drink, convulsions, lethargy or persistent vomiting), informed consent granted by parents of legal guardians. Exclusion criteria were i) haemoglobin < 5 g/dL; ii) severe conditions, such as prostration, respiratory distress, renal failure, hypoglycaemia, shock, bleeding, severe vomiting; and iii) a history of allergy or other severe adverse reaction to the study drugs.

Block-randomization was used to allocate patients to the three treatment arms (SP + AS, SP + AQ or SP). Treatment assignments were done through sequentially numbered opaque envelops that concealed the actual treatment to which the patient was randomized. The study statistician generated the randomization code using a computer system. The study microscopists assessing malaria smears were kept blinded throughout the study duration.

Enrolled patients were followed-up for 28 days. They were asked to return for clinical and/or biological follow-up on days 1, 2, 3, 7, 14, 21, 28, or on days of recurrent illness. Patients or guardians were asked about drug consumption since the last clinic visit, and about any adverse event. At each visit, a brief physical examination, including axillary temperature, was performed and blood was taken for thick smears. Patients were asked to return to the clinic if they became ill outside of regular visit times. Cases of treatment failures were treated with quinine. Patients failing to report to the clinic for two consecutive scheduled visits were considered lost to follow up.

### Treatments procedures

Arm 1 received SP + AS and arm 2 received SP + AQ, both in a loose combination and arm 3 received SP alone. Treatment was administered according to body weight at the following doses: AQ (Flavoquine®, Sanofi Aventis), 10 mg/kg/day on days 0, 1 and 2; AS (Arsumax®, Sanofi Aventis), 4 mg/kg/day on days 0, 1 and 2; SP, 25 mg/kg of sulphadoxine and 1.25 mg/kg of pyrimethamine (Fansidar®, Roche) in single dose on day 0. All drugs were administered directly by the study team at the respective health centres. Thereafter, each child was observed for 60 minutes. If vomiting occurred within 30 minutes, the dose was repeated; for vomiting after 30 minutes, a half-dose was administered. In the case of persistent vomiting (3 or more consecutive vomiting incidents), the child received a rescue treatment with intravenous quinine and withdrawn from the study. After the day of enrolment (day 0), patients were assessed on days 1, 2, 3, 7, 14, 21 and 28 using the same procedures described above.

### Laboratory procedures

Capillary blood was obtained by fingerpick; thick and thin blood films were made and stained with 5% Giemsa for 20 minutes. Parasite densities were determined from thick blood smears by counting the number of asexual parasites per 300 WBCs assuming a WBC count of 7,500/μl [19]. The same method was used to estimate gametocyte density but on 1,000 WBCs. A microscopist who was blinded to the treatment allocation read slides. A negative blood film was defined as a slide with no parasites after the review of 100 fields. Thin blood films were read only for parasite species identification. A second reader, blinded to the initial results for quality assurance, read ten percent of slides. A third microscopist read discordant slides. A discrepancy in the results between the two microscopists was defined as a positive/negative slide, a difference in species diagnosis, or >25% difference in parasite density. Each final parasite density was computed by averaging the two most concordant counts. For each positive/negative slide, the third reading was taken as the final result. Blood haemoglobin concentration was measured using a portable photometer (HemoCue: Anglholm, Sweden) on days 0, 14, 28 and any day of failure. Anaemia was defined as haemoglobin <11 g/dL.

To distinguish recrudescent from new infections, molecular genotyping of the parasite merozoite surface protein 2 (*msp2*) genes was used on samples from patients who failed after day 7. At enrolment and during the follow-up visits or unscheduled visit (with positive *P. falciparum* malaria), blood spots were obtained on filter papers (3MM Whatman) for molecular analysis.

DNA was extracted using the “methanol method” [20]. Samples that failed to yield interpretable results were re-extracted using a Qiagen® genomic DNA extraction kit according to the manufacturer’s instructions. Paired dried blood spots (from day 0 and the day of parasitaemia recurrence) were analysed by nested PCR [21,22]. Possible outcomes were: (i) recrudescence, if the *MSP2* alleles of the pre- and post-treatment samples were the same; (ii) re-infection, if the alleles of the pre- and post-treatment samples were distinct; (iii) mixed recrudescence and re-infection, if similar alleles were found in the pre- and post-treatment samples, but with additional distinct alleles identified; (iv) indeterminate, if either or both the pre- and post-treatment samples could not be amplified.

### Study endpoint classification

Treatment outcomes were classified following the WHO anti-malarial drug efficacy guidelines 2003 as early treatment failure (ETF), late clinical failure (LCF), late parasitological failure (LPF), and adequate clinical and parasitological response (ACPR) [18]. A per protocol analysis was used. Outcome measures were efficacy rates at day 28 before PCR adjustment (ACPR) or after PCR adjustment (cACPR). Other outcome measures were anaemia (haemoglobin <11.0 g/dL) as well as fever, parasite, and gametocyte clearance times.

### Sample size

The required sample size was calculated using the non-inferiority assumption that the three study drugs were equally effective in terms of the primary endpoint, with a two-sided α of 0.05 and a power of 85%. The maximum accepted difference in efficacy between the two study treatments was set at 5%. On the basis of an efficacy estimate of 95% on day 28, a total of 900 subjects were necessary (300 in each arm including the 10% lost during follow-up). No interim analysis was planned.

### Data entry and analysis

Data were double entered using Microsoft ACCESS and statistical analysis was performed using SPSS version 11.0 (Chicago, IL, USA). Baseline characteristics of subjects among groups were compared using the Fisher Exact Test and Chi-square tests for categorical variables and the Mann-Whitney *U*-test for non-normally distributed continuous variables. The proportion of patients failing treatment was compared across groups using the Chi-square test. The paired t-test was used to estimate the increase in haemoglobin content from baseline to different time-points of regular follow-up. Statistical significance was set at *p* < 0.05.

## Results

### Participant characteristics

**Figure 1:**
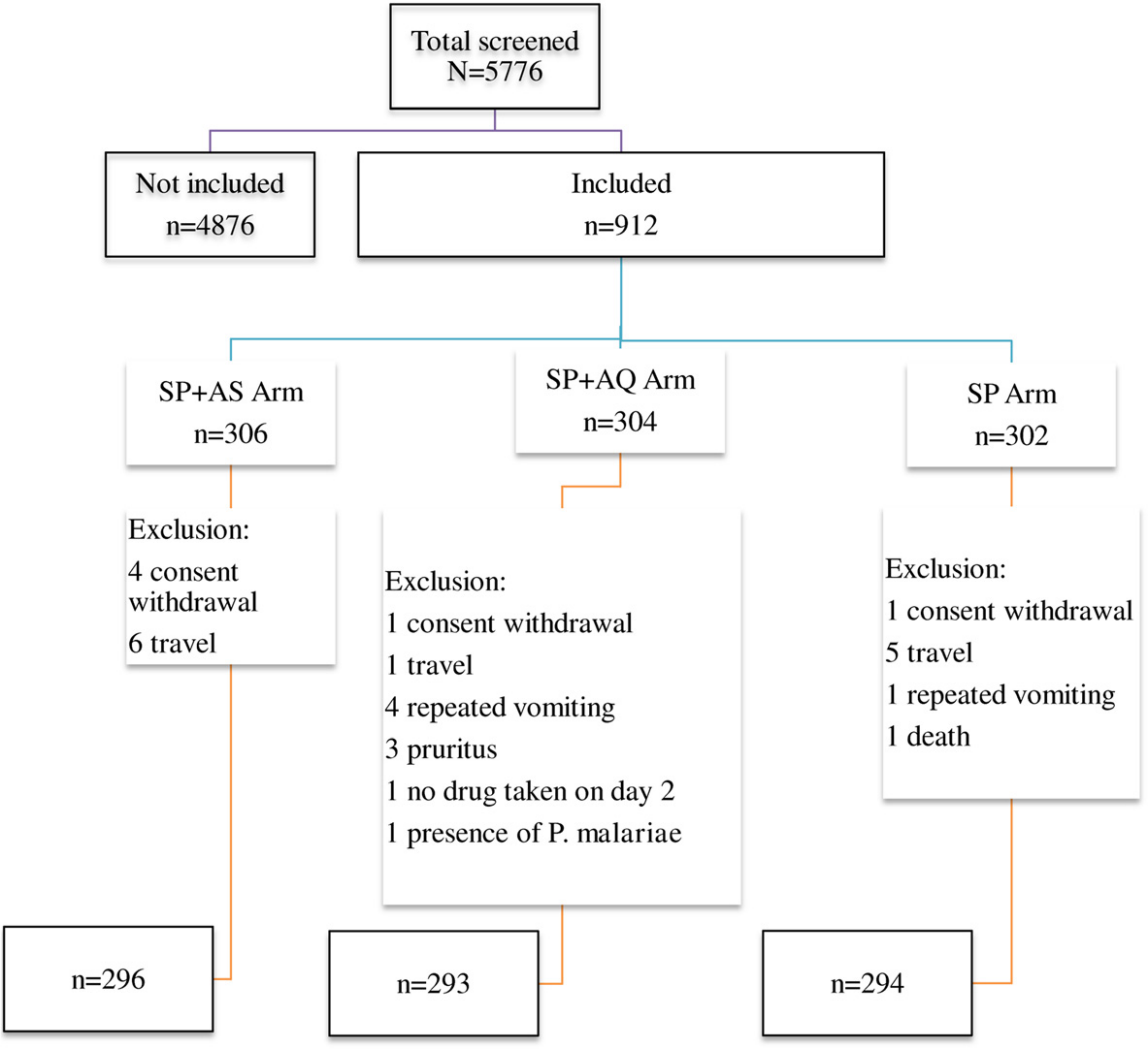
**Study profile.**

The trial profile is summarized in Figure 1. Overall 912 children aged between six to 59 months were enrolled in the study and assigned to treatment arms (Figure 1). Reasons for exclusion are shown in Figure 1. At day 28, 883 (296; 293 and 294) children were evaluated in SP + AS; SP + AQ and SP alone treatment arms, respectively. During follow-up, 29 children were unavailable for further study investigation (Figure 1). There was no difference between arms regarding the rate of loss to follow-up (p > 0.05). Baseline characteristics were similar for the three treatments arms (Table 1).

**Table 1:** **Demographic, clinical, and laboratory characteristics of patients at enrolment**

**Patient parameters**	**SP + AS**	**SP + AQ**	**SP**	***p-value***
	**(n = 306)**	**(n = 304)**	**(n = 302)**	
Sex (% male)	51.3	51.6	51.8	0.99
Weight (mean, Kg)	14.6	14.8	14.9	0.84
Age (mean, years)	3.9	4.1	4.2	0.62
Height (mean, cm)	94.7	96.0	96.5	0.54
Temperature (mean, °C)	38.4	38.4	38.4	0.37
*P. falciparum* parasite (mean, count/μL)	43,940	42,249	41,419	0.65
Gametocyte carriage (%)	11.4	9.5	12.6	0.48
Haemoglobin concentration (mean, g/dL)	10.6	10.6	10.7	0.61

### Cure rates

There were 1.4% (n = 294) early treatment failures for SP alone but none in the SP + AS and SP + AQ arms (Table 2). Crude ACPRs were 94.9%, 98.6% and 93.5% for SP + AS, SP + AQ and SP arms, respectively (SP + AS *versus* SP + AQ, p = 0.01; SP + AS *versus* SP, p = 0.5; SP + AQ *versus* SP, p = 0.001) (Table 2). After PCR adjustment, cACPRs were 99%; 100% and 97.2% for SP + AS; SP + AQ and SP arms, respectively (SP + AS *versus* SP + AQ, p = 0.25; SP + AS *versus* SP, p = 0.12; SP + AQ *versus* SP, p = 0.007) (Table 2).

**Table 2:** **ACPRs before and after molecular correction at Day 28**

	*ETF (%)	**LCF (%)**	**LPF (%)**	**ACPR (%)**	**cACPR (%)**
**SP + AS** (n = 296)	0.0	1.7	3.4	94.9	99.0
**SP + AQ** (n = 293)	0.0	0.0	1.4	98.6	100
**SP** (n = 294)	1.4	1.0	4.1	93.5	97.2
***p-value***				**a**	**b**

*see text for definitions.

a. χ^2^ *p* = 0.01 SP + AS *versus* SP + AQ; *p* = 0.5 SP + AS *versus* SP; *p* = 0.001 SP + AQ *versus* SP.

b. χ^2^ *p* = 0.25 SP + AS *versus* SP + AQ; *p* = 0.12 SP + AS *versus* SP; *p* = 0.007 SP + AQ *versus* SP.

### Fever clearance, parasite clearance, gametocyte carriage and anaemia

**Figure 2:**
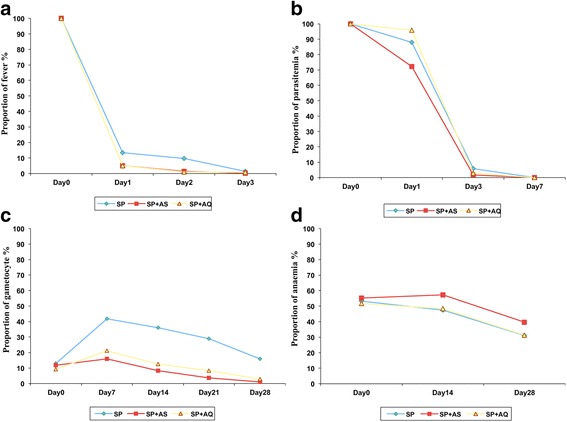
**Proportion of patients with fever (a), parasitaemia (b), gametocytes (c) and anaemia (d) at baseline to follow-up visit.**

As shown on Figure 2a, there was a rapid decline in fever prevalence in all three treatment arms. At Day 3, 1.7%; 2.6% and 5.9% of patients were still carrying malaria parasite in the SP + AS; SP + AQ and SP arms, respectively (p < 0.01 for SP + AS *versus* SP, no statistically significant difference for other comparisons) (Figure 2b). Using McNemar paired Chi-square test, gametocyte carriage significantly decreased between baseline and day 28 for SP + AS (11.8% *versus* 2.8%, p < 0.001) and SP + AQ (9.2% *versus*. 1%, p < 0.001). No such decrease was found for the SP alone arm (Figure 2c). All three-treatment arms significantly reduced anaemia prevalence by day 28 (SP + AS, 55.3% *versus* 39.7%; SP + AQ, 51.7% *versus* 31.2%; SP, 53.2% *versus* 31%; p < 0.001) (Figure 2d).

### Adverse events

SP + AS, SP + AQ and SP alone were well tolerated. The number of patients reporting any symptoms or signs within the first week after treatment began was statistically similar between the three treatment arms (Figure 1). There was one death in the SP arm of the study.

## Discussion

This study found that the non-artemisinin-based combination of SP + AQ was as efficacious as an artemisinin-based combination of SP + AS for the treatment of uncomplicated *P. falciparum* malaria in children less than five years of age. After PCR correction, the highest cACPR was observed with SP + AQ although the difference was not significant. Although these data are now over a decade old, they are similar to another study from a different site in Mali [23] and also corroborate those from other countries, which show equal or superior efficacy of SP + AQ when compared with AS + SP [24]. The data suggest that SP + AQ provided greater protection against new infections during the 28-day follow-up period than either SP or SP + AS. The prevention of new infections provided by SP + AQ is probably due to the long elimination half-lives of the component drugs [25]. In SP + AS, the artemisinin derivative on the other hand is quickly eliminated, yielding less post-treatment prophylaxis. Children treated with SP + AS or SP + AQ and SP alone had a lower prevalence of anaemia on days 14 to 28. Anaemia prevalence closely followed parasitaemia prevalence suggesting that the combination of two longer half-life drugs in SP + AQ provides better protection against new infections and subsequent anaemia.

The artemisinin-based combination, SP + AS, showed faster parasite clearance than SP + AQ, but both regimens resulted in prompt fever reduction. Despite the lower proportion of fever and parasitaemia observed during the first three days after treatment in the ACT arm (SP + AS), there were more new infections in this group than in the non-ACT arm (SP + AQ). Other studies have made similar observations. For example, in Rwanda [26], the United Republic of Tanzania [27] and Central African Republic, at least 25% resistance of *P. falciparum* to SP was found, but assessment of the clinical efficacy of combinations containing SP showed adequate clinical and parasitological response rates of 90.3% [28], 94% [27] and 100% [29] in the three countries, respectively. These results were significantly superior to earlier reports where cACPRs on day 28 for SP and AQ + SP were 70.1% and 80.9%, in Garoua, 62.5%, and 81.9% in Mutengene, and 67.5% and 76.2% in Yaoundé, respectively [30]. Both combination regimens were similar in the secondary endpoint of gametocyte carriage.

The Malian Ministry of Health is providing AS + AQ and AL free to children less than five years of age. Widespread implementation of ACT in this vulnerable population should produce a significant reduction in malaria mortality and morbidity in Mali. However, it is essential to continue monitoring the efficacy of this regimen, and to consider alternatives should evidence of resistance develop. Intermittent preventive treatment of children with SP + AQ, given during the malaria transmission season provided substantial protection against clinical episodes of malaria, malaria infection, and anaemia in children using an Long Lasting Insecticidal Nets (LLIN). As was found in this study, SP + AQ was shown to be safe and well tolerated [31,32]. These findings have now been translated into the new seasonal malaria chemoprevention (SMC) policy by World Health Organization [33]. As SMC with SP + AQ is currently being scaled up across the Sahel sub-region in Africa, the one worry is the potential development of resistance to these molecules, which needs to be regularly assessed.

## Conclusions

Sulphadoxine-pyrimethamine + amodiaquine therapy was as efficacious as sulphadoxine-pyrimethamine + artesunate, but more efficacious than sulphadoxine-pyrimethamine alone in the treatment of uncomplicated *P. falciparum* malaria in Mali. This study further supports the use of SP + AQ for SMC and of SP for IPTp in West Africa.

## Abbreviations

ACT: Artemisinin-based combination therapy
AQ: Amodiaquine
AS: Artesunate
CI: Confidence interval
PCR: Polymerase chain reaction
SP: Sulphadoxine-pyrimethamine
WBC: White blood cell count
WHO: World Health Organization
SMC: Seasonal malaria chemoprevention
IPTp: Intermittent preventive treatment of malaria during pregnancy
